# Release of metal ions from round and rectangular NiTi wires

**DOI:** 10.1186/s40510-016-0123-3

**Published:** 2016-04-04

**Authors:** Arash Azizi, Abdolreza Jamilian, Francesca Nucci, Zinat Kamali, Nima Hosseinikhoo, Letizia Perillo

**Affiliations:** Department of Oral and Maxillofacial Medicine, Tehran Dental Branch, Craniomaxillofacial Research Center, Islamic Azad University, Tehran, Iran; Department of Orthodontics, Tehran Dental Branch, Craniomaxillofacial Research Center, Islamic Azad University, No 2713, Vali Asr St., Tehran, 1966843133 Iran; Dental School, Universidad Alfonso X El Sabio, Madrid, Spain; National Nutrition and Food Technology Research Institute, Faculty of Nutrition Sciences and Food Technology, Shahid Beheshti University of Medical Sciences, Tehran, Iran; Tehran Dental Branch, Islamic Azad University, Tehran, Iran; Multidisciplinary Department of Medical-Surgical and Dental Specialties, Second University of Naples, Naples, Italy

## Abstract

**Background:**

The aim of this study was to evaluate the amount of nickel and titanium ions released from two wires with different shapes and a similar surface area.

**Methods:**

Forty round nickel-titanium (NiTi) arch wires with the diameter of 0.020 in. and 40 rectangular NiTi arch wires with the diameter of 0.016 × 0.016 in. were immersed in artificial saliva during a 21-day period. The surface area of both wires was 0.44 in.^2^. Wires were separately dipped into polypropylene tubes containing 50 ml of buffer solution and were incubated and maintained at 37 °C. Inductively coupled plasma atomic emission spectrometry (ICP-AES) was used to measure the amount of ions released after exposure lengths of 1 h, 24 h, 1 week, and 3 weeks. Repeated measures ANOVA and Tukey tests were used to evaluate the data.

**Results:**

The results indicated that the amount of nickel and titanium concentrations was significantly higher in the rectangular wire group. The most significant release of all metals was measured after the first hour of immersion. In the rectangular wire group, 243 ± 4.2 ng/ml of nickel was released after 1 h, while 221.4 ± 1.7 ng/ml of nickel was released in the round wire group. Similarly, 243.3 ± 2.8 ng/ml of titanium was released in the rectangular wire group and a significantly lower amount of 211.9 ± 2.3 ng/ml of titanium was released in the round wire group.

**Conclusions:**

Release of metal ions was influenced by the shape of the wire and increase of time.

## Background

Orthodontic appliances are made of alloys containing nickel, cobalt, and chromium in different percentages. Thermal, microbiologic, and enzymatic properties of the oral environment make it an ideal place for the biodegradation of these alloys. Metals used as components of these alloys, i.e., nickel and chromium, have been identified as cytotoxic, mutagenic, and allergenic [1]. In addition, nickel is the most common cause of metal-induced allergic contact dermatitis and produces more allergic reactions than all other metals combined, followed by chromium [2]. It has been shown that the level of nickel in saliva and serum increases significantly after the insertion of fixed orthodontic appliances [3]. Nickel-titanium (NiTi) wire is one of the most common orthodontic wires applied clinically due to its good working and mechanical properties; however, nickel-titanium wires may contain in excess of 50 % nickel, while stainless steel wires contain only 8 % nickel [4–6].

Previous studies have investigated, both in vitro and in vivo, release of metal ions from orthodontic appliances [3, 7–11]. Mikulewicz et al. [12] found that the use of fixed orthodontic appliances made of stainless steel can be a source of risk exposure to nickel. Huang et al. [13] reported that the manufacturer, pH, and immersion period had a statistically significant influence on the release amount of nickel and titanium ions from commercial NiTi wires in acidic artificial saliva. Kuhta et al. [14] studied the effects of wire type on the release of metal ions and found that release of metal ions was influenced by composition of the orthodontic arch wire, but this was not proportional to the content of metal in the wire. An electronic search in the literature reveals that no information exists about the effects of different cross sections of different wires on the release of Ni and Ti ions. The aim of this study was, therefore, to compare the effects of round and rectangular NiTi wires and different immersion times on the release of Ni and Ti ions in artificial saliva.

## Methods

Eighty orthodontic NiTi arch wires with round and rectangular cross sections were used in this study (Ortho Technology, Tampa, FL, USA). Both wires were 7 in. long. Group 1 consisted of 40 round NiTi arch wires with the diameter of 0.020 in., and group 2 included 40 rectangular NiTi arch wires with the diameter of 0.016 × 0.016 in. The surface area of both wires was 0.44 in.^2^. Each group was divided into four equal subgroups for four different time points. The testing solution used in the present study was artificial saliva (Sinphar Pharmaceutical Co., Ltd, Taipei, Taiwan) which was adjusted with 1 mM NaHNO_3_ to pH 6.75 ± 0.15 buffer solutions (Table 1). Wires were separately dipped into polypropylene tubes containing 50 ml of buffer solution and were incubated at 37 °C and placed on a shaker to simulate in vivo situation. In this study, we measured the amount of ion released after exposure lengths of 1 h, 24 h, 1 week, and 3 weeks.

**Table 1:** Content of SaliLube (Sinphar Pharmaceutical Co., Ltd, Taipei, Taiwan) artificial saliva

Content	Amount (mg)
Sodium chloride	0.844
Potassium chloride	1.2
Calcium chloride anhydrous	0.146
Magnesium chloride 6 H_2_O	0.052
Potassium phosphate dibasic	0.34
Sorbitol solution (70 %)	60
Methylparaben	2
Hydroxyethyl cellulose	3.5

At the end of each immersion period, 5 ml of eluent was removed from each solution using a syringe with a plastic tip. Inductively coupled plasma atomic emission spectrometry (ICP-AES) was used to determine the amount of Ni and Ti ions released after each immersion period.

### Statistical analyses

The release amount of Ni and Ti ions at different time points for different cross sections was statistically analyzed by repeated measures ANOVA. Tukey grouping method was used to compare the concentration of Ni and Ti released for the different time points and types of wires. All results were analyzed at a significance level of 5 %.

## Results

Tables 2 and 3 show the accumulated amount of Ni and Ti ions released from rectangular and round NiTi wires after different immersion periods of 1 h, 24 h, 1 week, and 3 weeks. The results of the Kolmogorov-Smirnov test showed that all ions had normal distributions.

**Table 2:** Release of nickel in rectangular and round NiTi wires at different time points

Time	Rectangular wire	Round wire	*P* value
	Mean ± SD	Mean ± SD	
1 h	243 ± 4.2	221.4 ± 1.7	0.001
24 h	319.4 ± 2.9	265.1 ± 2.7	0.001
1 week	386.1 ± 3	324.2 ± 3.6	0.001
3 weeks	410.6 ± 2.6	376 ± 3	0.001
*P* value	0.001	0.001	0.001

**Table 3:** Release of titanium in rectangular and round NiTi wires at different time points

Time	Rectangular wire	Round wire	*P* value
	Mean ± SD	Mean ± SD	
1 h	243.3 ± 2.8	211.9 ± 2.3	0.001
24 h	288.4 ± 5	253.7 ± 3.9	0.001
1 week	318.8 ± 1.6	290 ± 1.6	0.001
3 weeks	385.6 ± 4.4	329.5 ± 6.3	0.001
*P* value	0.001	0.001	0.001

After 1 h of immersion, 243 ± 4.2 ng/ml nickel was released from rectangular wires in the artificial saliva while 221.4 ± 1.7 ng/ml nickel was released from round wires (*P* < 0.001). Repeated measures ANOVA showed that in all immersion periods, the amount of nickel and titanium released from rectangular wires was significantly higher than the ions released from round wires.

A comparison of nickel and titanium release from both rectangular and round wires at different immersion periods showed that, in all groups, the accumulated amount of metal ions increased with immersion period while the average ion released per day decreased with immersion period. Repeated measures ANOVA showed that the increase of released ions at different immersion periods was statistically significant for all of them (*P* < 0.001) (Tables 2 and 3). In addition, Tukey test showed that there has been a significant difference between all the time intervals in each group.

## Discussion

The aim of this study was to determine the nickel and titanium release from two different shapes of NiTi wires by immersion of the samples in artificial saliva. The wires had different cross sections but the same surface area. The cross sections were round and rectangular. In order for the rectangular and round NiTi wires to have the same surface area, 0.020-in. round and 0.016 × 0.016 in. rectangular wires were used in this study. The current study showed that with the same surface area, the concentration of Ni and Ti ions transferred from rectangular wires to the saliva is significantly higher than that of round wires. The difference might be due to the fact that cylindrical and rectangular bar shapes with the same surface areas do not have the same volume. The difference might also be due to the different edges of the cross sections. Further research is required to provide evidence for the effect of sharp and round edges on the release of ions into the saliva. The current study also showed that the amount of Ni and Ti ions significantly increased in both groups with the passage of time.

Ni is a widespread component of the Earth’s surface. Its presence in food and drinking water is determined by both natural and anthropogenic factors, the latter generically identifiable with industrial and technological sources. Ni and Ni compounds have been classified as carcinogenic to humans causing cancers of the lung and nasal cavity and paranasal sinuses after inhalation.

Numerous in vitro and in vivo studies have evaluated the release of metals from orthodontic appliances in biologic fluids such as saliva, blood, and urine.

Kuhta et al. [14] analyzed the effects of pH, composition of arch wire, and length of immersion on the release of metal ions from orthodontic appliances and found that release of metal ions was influenced by composition irrespective to the content of metal in the wire. Wataha et al. [15] also reported that although NiTi wires have a high percentage of nickel, the quantity of released nickel ions is smaller than that released from SS wire.

Most studies reported that the measurable amount of metals, released from orthodontic appliances in saliva or blood samples, was significantly below the toxic concentrations [3, 16]. Senkutvan et al. [17] analyzed the rate of Ni ion release from different types of arch wires used in orthodontics. They found that the quantities of metal ions released in their experimental conditions were not a concern in utilizing the appliance as the amount released was below the critical value necessary to induce allergy and below the daily dietary intake levels of 200–300 μg/day. Nonetheless, it cannot be excluded that even nontoxic concentrations might be sufficient to induce important biologic effects in the cells of the oral mucosa [9].

The release of metal ions from orthodontic appliances cannot be fully avoided; however, it would be advisable to use materials with lower amounts of ions released in the mouth. If the clinician has the option of using either round or rectangular NiTi wires, we suggest that round wires be given priority. However, more in vivo experiments are required to firmly determine the levels of dissolved nickel and titanium in different shapes of wires. Future studies with a lower pH to simulate intraoral conditions present with plaque buildup are also advisable. Comparing the surface roughness between round and rectangular wires from different manufacturers can also be enlightening for clinicians.

## Conclusions

- The release amount of Ni and Ti ions increased with immersion period while the average ion released per day decreased with immersion period.
- The amounts of Ni and Ti ions released from rectangular NiTi wires in artificial saliva were greater than those in round wires; however, the average amount of ions released per day from both wires was well below the tolerable daily dietary intake level.
